# Malaria Vector Bionomics: Countrywide Surveillance Study on Implications for Malaria Elimination in India

**DOI:** 10.2196/42050

**Published:** 2024-06-17

**Authors:** Manju Rahi, AK Mishra, Gyan Chand, RK Baharia, RK Hazara, SP Singh, Siraj Khan, U Sreehari, Divya Kamaraju, Gaurav Kumar, Sanjeev Kumar Gupta, Amit Sharma, K Raghavendra, K Gunasekaran, Om P Singh, Sarala K Subbarao

**Affiliations:** 1 Indian Council of Medical Research Delhi India; 2 Academy of Scientific and Innovation Research Ghaziabad, Uttar Pradesh India; 3 Vector Control Research Centre Puducherry India; 4 National Institute of Research in Tribal Health Jabalpur, Madhya Pradesh India; 5 National Institute of Malaria Research New Delhi India; 6 Regional Medical Research Centre Bhubaneswar, Odisha India; 7 Regional Medical Research Centre Dibrugarh, Assam India; 8 Molecular Medicine Group International Centre of Genetic Engineering and Biotechnology New Delhi India

**Keywords:** malaria, bionomics, sibling species, insecticide resistance, elimination, India

## Abstract

**Background:**

The biological characteristics of mosquito vectors vary, impacting their response to control measures. Thus, having up-to-date information on vector bionomics is essential to maintain the effectiveness of existing control strategies and tools, particularly as India aims for malaria elimination by 2030.

**Objective:**

This study aims to assess the proportions of vector species resting indoors and outdoors, determine their preference for host biting/feeding, identify transmission sites, and evaluate the susceptibility of vectors to insecticides used in public health programs.

**Methods:**

Mosquito collections were conducted in 13 districts across 8 Indian states from 2017 to 2020 using various methods to estimate their densities. Following morphological identification in the field, sibling species of Anopheles mosquitoes were identified molecularly using polymerase chain reaction (PCR)–specific alleles. Plasmodium falciparum and Plasmodium vivax infections in the vectors were detected using enzyme-linked immunosorbent assay (ELISA) and PCR assays. In addition, we assessed the insecticide susceptibility status of primary malaria vectors following the World Health Organization (WHO) protocol.

**Results:**

Anopheles culicifacies, a primary malaria vector, was collected (with a man-hour density ranging from 3.1 to 15.9) from all states of India except those in the northeastern region. Anopheles fluviatilis, another primary vector, was collected from the states of Madhya Pradesh, Maharashtra, Karnataka, and Odisha. In Haryana and Karnataka, An. culicifacies sibling species A predominated, whereas species C and E were predominant in Madhya Pradesh and Maharashtra. An. culicifacies displayed mainly endophilic behavior across all states, except in Madhya Pradesh, where the proportion of semigravid and gravid mosquitoes was nearly half of that of unfed mosquitoes. The human blood index of An. culicifacies ranged from 0.001 to 0.220 across all study sites. The sporozoite rate of An. culicifacies ranged from 0.06 to 4.24, except in Madhya Pradesh, where none of the vector mosquitoes were found to be infected with the Plasmodium parasite. In the study area, An. culicifacies exhibited resistance to DDT (dichlorodiphenyltrichloroethane; with <39% mortality). Moreover, it showed resistance to malathion (with mortality rates ranging from 49% to 78%) in all districts except Angul in Odisha and Palwal in Haryana. In addition, resistance to deltamethrin was observed in districts of Maharashtra, Gujarat, Haryana, and Karnataka.

**Conclusions:**

Our study offers vital insights into the prevalence, resting behavior, and sibling species composition of malaria vectors in India. It is evident from our findings that resistance development in An. culicifacies, the primary vector, to synthetic pyrethroids is on the rise in the country. Furthermore, the results of our study suggest a potential change in the resting behavior of An. culicifacies in Madhya Pradesh, although further studies are required to confirm this shift definitively. These findings are essential for the development of effective vector control strategies in India, aligning with the goal of malaria elimination by 2030.

## Introduction

Malaria continues to stand as a significant contributor to both morbidity and mortality within the realm of vector-borne diseases. As outlined in the World Malaria Report of 2021, there were 241 million reported cases and 627,000 deaths attributed to malaria, with the majority occurring in African nations (accounting for 95% of the total cases) [1]. The World Health Organization South-East Asia Region (WHO-SEARO) contributed approximately 2% to the global malaria burden. Over the past 2 decades, malaria incidence in this region has seen a notable decline, dropping from 23 million cases in 2000 (18 cases per 1000) to approximately 5 million cases in 2020 (3 cases per 1000). India has shown remarkable progress within the WHO-SEARO zone, achieving substantial reductions from approximately 20 million malaria cases in 2000 to around 4.1 million cases in 2020. However, despite this improvement, India still represented 83% of malaria cases and 82% of malaria fatalities in the South-East Asian region [1]. Data from the national program in 2020 further indicated that approximately 82% of malaria cases reported in India were concentrated in 6 states: Odisha, Chhattisgarh, Uttar Pradesh, Jharkhand, Maharashtra, and West Bengal [2].

The significant reduction in the malaria burden across India can be largely attributed to the expansion of control interventions. These include prompt diagnosis facilitated by the widespread availability of rapid diagnostics and microscopy, treatment using artemisinin-based combination therapy, and crucially, the effective implementation of vector control measures such as indoor residual spraying (IRS) and distribution of long-lasting insecticidal nets (LLINs). In India, 6 primary malaria vectors have been identified, namely, *Anopheles culicifacies, Anopheles fluviatilis, Anopheles minimus*, *Anopheles stephensi, Anopheles baimaii,* and *Anopheles sundaicus*. The secondary vectors are *Anopheles annularis, Anopheles philippinensis, Anopheles nivipes,* and *Anopheles varuna* [3]. According to national guidelines, the distribution of LLINs aims for 80% coverage, with an average of 1 LLIN per 1.8 people, particularly targeting all subcenters reporting an annual parasite incidence of greater than 1. IRS, by contrast, is directed toward epidemic-prone areas and malaria-affected communities with limited access to health care services [4]. Vector control interventions have played a pivotal role and have been the primary contributors to the reduction in malaria transmission.

In the pursuit of malaria elimination, the effectiveness of vector control interventions hinges greatly on understanding the diverse biological characteristics of vectors [5]. These include physiological and behavioral traits that directly impact malaria transmission. Therefore, to maintain the efficacy of vector control measures, it is imperative to prioritize the entomological aspects of prevalent malaria vectors. This entails staying abreast of recent data on their bionomics and assessing the efficacy of chemical interventions. Such insights are crucial for evaluating the effectiveness of current vector control tools and for making informed decisions regarding the most suitable control strategies to be deployed in the field.

India’s diverse topographies, climatic conditions, and ecosystems give rise to varying levels of malaria endemicity across the country [6]. In addition to the presence of multiple vectors, India faces the challenge of vector incursion and migration. *An. culicifacies,* a primary malaria vector in India, has expanded its range into the northeastern states, where it has established itself as a malaria vector in this previously unaffected territory [7]. Likewise, *An. stephensi,* an urban malaria vector in India, has spread its presence into regions such as Sri Lanka, Djibouti, Ethiopia, and Sudan in Africa [8]. The emergence of these vectors in new areas highlights the critical need for continuous entomological surveillance to monitor and effectively address these evolving challenges. The migration of various malaria vectors across neighboring areas, ecological transitions, and colonization of new habitats through evolutionary adaptation to eco-climatic changes have been documented in numerous instances in India [9]. An entomological surveillance study [10] was conducted to monitor the *Aedes* invasion in the Guilan Province of Iran, which boasts several ports of entry. Although no specimens of *Aedes aegypti* or *Aedes albopictus* were collected, the importance of such regular surveillance was acknowledged [10]. Similarly, in India, the Indian Council of Medical Research’s (ICMR) Vector Control Research Centre is conducting a study to explore the relationship between human and mosquito mobility and its implications for the control of mosquito-borne infections. In this regard, this study aims to unravel the intricate phenomenon of dispersion and movement of short- and long-distance cargo, as well as human transportation, along with the surrounding travel infrastructure such as ports, stations, and markets. Recently, there has been an increasing demand for comprehensive nationwide vector surveillance studies [11]. The ICMR, headquartered in New Delhi, serves as the nodal research organization under the Ministry of Health and Family Welfare, tasked with conducting biomedical research across the country. The ICMR, through its permanent institutes located in several malaria-endemic states, has conducted extensive entomological research over the past several decades. It has made significant contributions to the understanding and dissemination of the science of vector biology in the country, particularly in the context of malaria elimination goals [12]. The current multicenter study was conceptualized and formulated by the ICMR. In consultation with experts, common objectives were formulated, and a standardized methodology for studying vector bionomics was developed. The study was conducted by 4 research institutes of the ICMR and their respective field stations situated across different regions of the country from 2017 to 2020. The objectives of the study were to assess (1) the proportions of vector species resting indoors and outdoors over time; (2) host biting/feeding preferences, biting rhythms, and peak biting activity of vector species across different seasons; (3) the sites of transmission; and (4) the susceptibility status of vectors to various insecticides.

## Methods

### Study Sites

**Figure 1:**
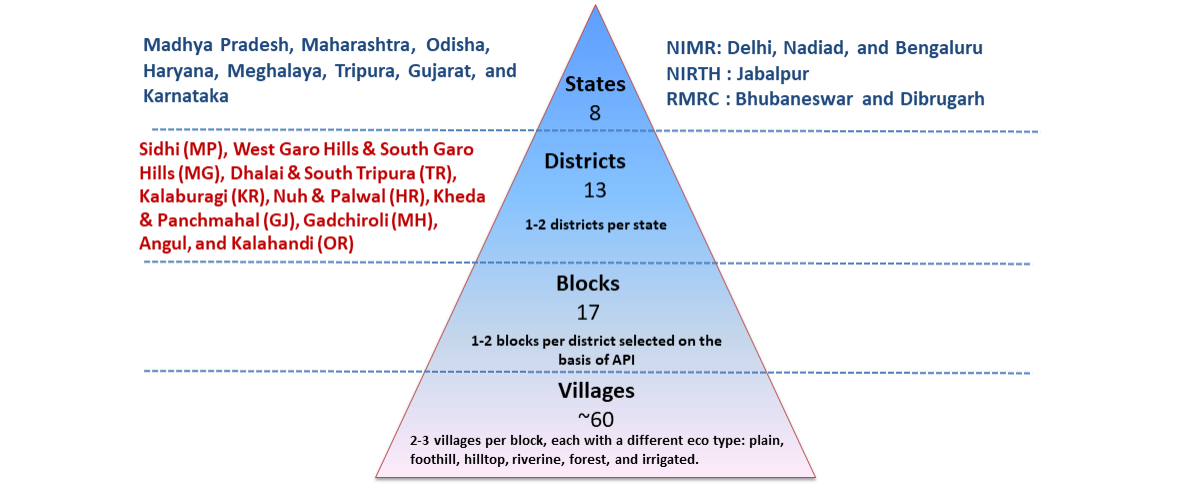
Criteria for study site selection. GJ: Gujarat; HR: Haryana; KR: Karnataka; MG: Meghalaya; MH: Maharashtra; MP: Madhya Pradesh; NIMR: National Institute of Malaria Research; NIRTH: National Institute for Research on Tribal Health; OR: Odisha; RMRC: Regional Medical Research Center; TR: Tripura.

**Figure 2:**
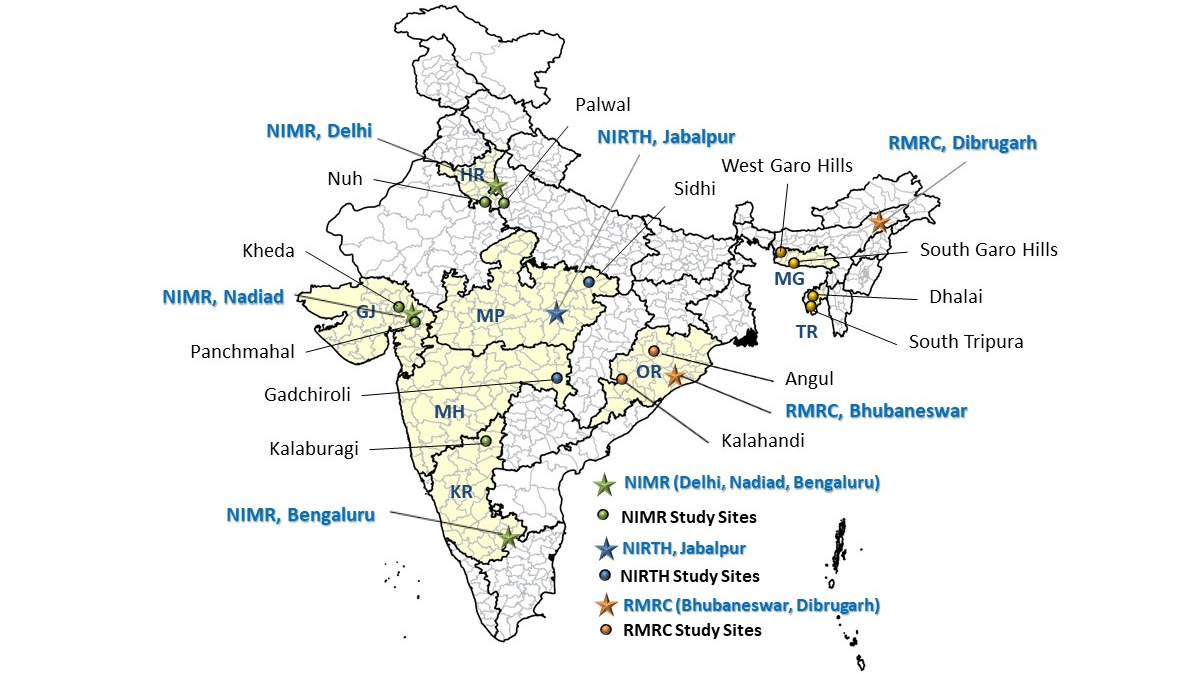
Location of participating institutes and study sites. GJ: Gujarat; HR: Haryana; KR: Karnataka; MG: Meghalaya; MH: Maharashtra; MP: Madhya Pradesh; NIMR: National Institute of Malaria Research; NIRTH: National Institute for Research on Tribal Health; OR: Odisha; RMRC: Regional Medical Research Center; TR: Tripura.

The study was conducted by 4 institutes of the ICMR: the National Institute of Malaria Research in Delhi, along with its field units in Bengaluru and Nadiad; the National Institute of Research in Tribal Health in Jabalpur, Madhya Pradesh; the Regional Medical Research Centre in Bhubaneswar, Odisha; and the Regional Medical Research Centre in Dibrugarh, Assam. A total of 13 districts from 8 Indian states, each characterized by distinct ecotypes, vector distributions, insecticide susceptibility statuses, among others, were included in the study. In each district, we identified 1 or more blocks (subdistrict level) with varying epidemiological situations. Within each block, we selected 2-3 villages, each representing a characteristic ecotype such as forest, foothill, riverine, or plains. The study encompassed all 4 prevailing seasons: premonsoon, monsoon, postmonsoon, and winter. Figure 1 illustrates the criteria, number of states, districts, blocks/community health centers (CHCs), and villages selected for the study. Figure 2 depicts the locations of the participating institutes and the districts covered by each institute.

### Ethical Approval

After community sensitization, verbal approval was sought from the chiefs and elders of the communities for mosquito collection in the selected villages. In addition, written consent was obtained from individuals who participated as human baits. Given that the study protocol entailed human landing collections, which could potentially result in mosquito bites on the human baits, efforts were made to collect mosquitoes immediately upon landing on the host, before engorgement, to prevent actual biting. The human baits received prophylaxis as part of the malaria prevention measures. Ethical approval for the study was obtained from the ethics committee of each respective participating institute.

Institutional Ethical Committee approval was obtained vide letter no. ECR/NIMR/EC/2017/142 dated 21 June 2017 by National Institute of Malaria Research.

### Entomological Surveys

#### Overview

The various methods used for the collection of adult anopheline mosquitoes from the study sites are described in the following sections.

#### Indoor Resting Collections

Anopheline mosquitoes resting indoors were captured manually using a mouth aspirator and a flashlight. In each village, 4 human dwellings and 4 cattle sheds were searched for mosquitoes in the morning from 6 AM to 8 AM hours, allocating 15 minutes for each structure [13]. The standard morpho-taxonomic keys were used to identify the mosquito species collected from the field [14]. The density of each vector species, expressed as per man-hour density (MHD), was calculated from the number of female mosquitoes, as per the formula provided in the “Data Analysis” section.

#### Light Trap Collections (Center for Disease Control Light Trap)

Adult vector density was also monitored using light traps. These traps were suspended inside human dwellings near eaves, sleeping hosts, and doors for mosquito collection from 6 PM to 6 AM. Outdoor collections were conducted by hanging traps both near the houses and in open areas away from the houses. The collected mosquitoes were placed in cartons lined with wet towels at the bottom and kept outside during transportation to maintain a temperature of 26-28°C and a relative humidity of 70%-80%. Vector density, defined as the number of female mosquitoes collected per trap night indoors or outdoors, was calculated from the light trap collections.

#### Pyrethrum Spray Collection

Another method used in the study for collecting adult mosquitoes that were resting indoors was pyrethrum spray collection. Most, if not all, of the *Anopheles* mosquitoes resting indoors were collected using this method during the morning hours (8 AM to 10 AM). In this method, the entire floor of the room (human dwelling) was covered with a white cotton sheet. Using a flit sprayer, the complete room was sprayed with 0.1%-0.2% pyrethrum spray, causing all mosquitoes resting inside the room to be knocked down onto the sheet. The collected mosquitoes were then transferred into petri dishes lined with wet cotton or filter paper and transported to the laboratory. This method provided the total number of mosquitoes and species resting per house or structure.

#### Mosquito Landing Collections on Human Baits

A whole-night collection of mosquitoes landing on human bait was conducted from dusk to dawn (6 PM to 6 AM). Landing collections were made hourly, continuously for 12 hours. Mosquitoes were collected from the exposed legs of the human bait. Efforts were made to collect mosquitoes immediately upon landing on the host, before engorgement, to prevent actual biting. Mosquitoes collected from each hour were placed in separately labeled paper cups covered with a mosquito net. These collections were used for estimating the man-biting/landing density and determining the entomological inoculation rate of vector species.

### Laboratory Processing

#### Mosquito Species Identification

Mosquitoes collected through various methods were morphologically identified to different *Anopheles* species using the taxonomic keys provided by Nagpal and Sharma [14].

#### Sibling Species Identification

The mosquito specimens preserved in isopropanol underwent molecular identification to distinguish sibling species of *An. culicifacies* and *An. fluviatilis,* following the methods outlined by Goswami et al [15] and Singh et al [16], respectively.

#### Blood Meal Preferences

Blood from the stomachs of fully-fed mosquitoes obtained from the field was collected onto Whatman No. 1 filter paper to identify the source of the blood meal against human and bovine antisera. The human blood index (HBI) of *An. culicifacies* was determined using the gel diffusion technique [17].

#### Vector Incrimination

Mosquitoes collected via various methods were examined for vector infection with human malaria parasites. The heads and thoraces of the mosquitoes were dissected and stored in isopropanol at –20°C until use. Both polymerase chain reaction (PCR) and enzyme-linked immunosorbent assay (ELISA) were used as the primary methods for detecting sporozoites in mosquitoes, and both methods were applied in our study. Vector incrimination was conducted using the ELISA-based method to detect species-specific circumsporozoite antigen of *Plasmodium falciparum,* *Plasmodium vivax* 210, and *P. vivax* 247, following the protocol outlined by Wirtz et al [18,19]. In addition, diagnostic PCR was used for the detection of malaria parasites. In our study, a total of 4067 *An. culicifacies* mosquitoes were tested by PCR, and 4164 *An. culicifacies* mosquitoes were tested by ELISA. In our study, specific antibodies against circumsporozoite protein were used in ELISA, enabling the identification of *Plasmodium* parasite species as well as the subtyping of *P. vivax* sporozoites. Furthermore, PCR was conducted using the nested PCR protocol outlined by Snounou et al [20].

#### Insecticide Susceptibility Tests

Vector susceptibility to the insecticides used in the national control program was assessed once during the peak abundance of vector species, adhering to the guidelines set by the WHO [21]. Field-collected, preferably from unsprayed villages or houses, mixed-age vector mosquitoes were exposed to WHO papers impregnated with insecticides at diagnostic concentrations (DDT [dichlorodiphenyltrichloroethane] 4%, malathion 5%, and WHO-recommended discriminating concentrations of various synthetic pyrethroids) using WHO test kits. For each test, 100 mosquitoes were exposed in 4 or 5 replicates, with 20-25 mosquitoes per replicate for the treatment group and 50 mosquitoes in 2 replicates, with 25 mosquitoes per replicate, for the control group. The tests were conducted in a room free from insecticide contamination and maintained at a temperature of 26-28°C and a relative humidity of 70%-80% both during exposure and the subsequent 24-hour holding period. Following the 24-hour holding period, the percent mortality was calculated based on the total number of alive and dead mosquitoes in the replicates. If the control mortality fell within the range of 5%-20%, the Abbott formula was applied to correct the treatment mortality. According to the criteria outlined by the WHO, mortality rates of 98% or higher were categorized as “susceptible,” mortality rates below 90% were classified as “resistant,” and mortality rates between 91% and 97% were labeled as “possible resistance.”

### Data Analysis

All data generated during the study were inputted into a computer using Microsoft Excel, and the following parameters were analyzed:

- MHD, calculated as the number of mosquitoes collected by 1 person in 1 hour. It is determined by considering the total number of mosquitoes (n) collected, the time spent in minutes (t), and the number of persons involved in the collection (p). The formula for MHD is MHD = n × 60/t × p.
- The HBI, calculated based on the proportion of fed *Anopheles* mosquitoes found to contain human blood.
- The sporozoite rate, calculated based on the proportion of female *Anopheles* mosquitoes carrying sporozoites in their salivary glands.
- The human landing density, defined as the ratio of the total mosquitoes captured landing on a human bait for a given period to the total person-nights used for the same period.
- The entomological inoculation rate, calculated from human landing/biting catches as the product of the human landing density and the sporozoite rate of mosquitoes.

## Results

### Anopheles Species Collection Methods and Results: Insights from Resting, Pyrethrum Spray, Light Traps, and Human Landing Surveys

The results of *Anopheles* species collected through various methods including resting, pyrethrum spray, light traps, and human landing at different sites are described below and summarized in Table 1.

**Table 1:** Malaria vector density by various collection methods in districts of India.

State and district			*Anopheles* species		Indoor resting collection (per man-hour)^a^		Pyrethrum spray (number per room)		Light trap outdoor (per trap per night)		Light trap indoor (per trap per night)		Human landing indoors (per man per night)		Human landing outdoors (per man per night)		Animal bait collection (per bait per night)
**Gujarat**																	
	Kheda	*An. culicifacies*		3.15		1.92		3.9		3.1		1.85		—^b^		—	
	Panchmahal	*An. culicifacies*		4		2.46		3.08		1.41		1.04		—		—	
**Madhya Pradesh**																	
	Sidhi	*An. culicifacies*		9.47		3.94		3.88		—		0.5		0.6		3.11	
**Haryana**																	
	Nuh	*An. culicifacies*		15.95		10.96		0.41		—		—		4.2		—	
	Palwal	*An. culicifacies*		15.46		36.03		1.02		—		—		3.3		—	
**Maharashtra**																	
	Gadchiroli	*An. culicifacies*		12.1		3.7		0.06		0.06		0		0		1.5	
**Karnataka**																	
	Kalaburagi	*An. culicifacies*		5.86		2.3		0.48		3.71		12		—		12	
**Odisha**																	
	Kalahandi	*An. culicifacies*		—		—		1.1		4.5		—		—		—	
	Angul	*An. culicifacies*		—		—		1		2		—		—		—	
**Gujarat**																	
	Kheda	*An. stephensi*		0.17		0.06		0		0		0		—		—	
	Panchmahal	*An. stephensi*		0.025		0.016		0		0		0.125		—		—	
**Haryana**																	
	Nuh	*An. stephensi*		8		20.44		2.92		—		—		4.5		—	
	Palwal	*An. stephensi*		21.39		45.03		8.5		—		—		4		—	
**Madhya Pradesh**																	
	Sidhi	*An. fluviatilis*		0.74		0.33		0.49		—		0.01		0.01		0.24	
**Karnataka**																	
	Kalaburagi	*An. fluviatilis*		0.06		0.06		2.96		18.6		22.4		—		22.7	
**Odisha**																	
	Kalahandi	*An. fluviatilis*		—		—		2.3		3.8		—		—		—	
	Angul	*An. fluviatilis*		—		—		0.5		1.2		—		—		—	

^a^Man-hour density is defined as the number of mosquitoes collected by 1 person in 1 hour.

^b^Not done.

### Density and Proportion of Indoor Resting Anopheline Vectors

#### Madhya Pradesh (Sidhi District)

**Figure 3:**
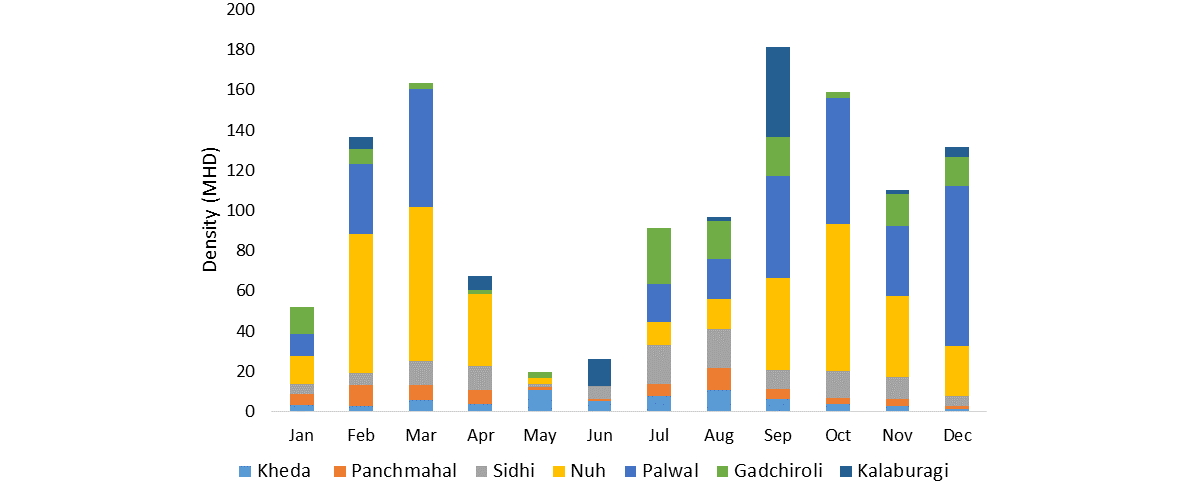
Monthly density of Anopheles culicifacies collected from selected districts of India. MHD: man-hour density.

*An. culicifacies* and *An. fluviatilis* vectors were prevalent, with an MHD of 9.47 (62%) and 0.47 (4%), respectively (Table 1). In addition, 13 other *Anopheles* species were collected, including *An. annularis,* a recognized secondary vector. The majority of *An. culicifacies* (92.3%) and *An. fluviatilis* (81%) were collected from cattle sheds. July and August recorded the highest density of anopheline mosquitoes, including the 2 vector species, whereas May and June had the lowest (Figure 3).

#### Gujarat (Kheda and Panchmahal Districts)

Three vector species, namely, *An. culicifacies, An. stephensi,* and *An. fluviatilis,* were collected. *An. culicifacies* predominated in Kheda and Panchmahal, with an MHD of 3.15 and 4.0, respectively. The MHD of *An. stephensi* and *An. fluviatilis* was less than 0.2. Further, 3 other *Anopheles* species, namely, *An. subpictus, An. vagus,* and *An. annularis,* were collected. The density of *An. culicifacies* was higher in cattle sheds in Panchmahal compared with that in Kheda. However, overall, anopheline density was higher in human dwellings in Kheda than in Panchmahal. Two peaks of *An. culicifacies* densities were observed in Gujarat, specifically in February-April and July-August.

#### Karnataka (Kalaburagi District)

In this area, *An. culicifacies, An. stephensi,* and *An. fluviatilis* were the 3 recorded vector species, while other *Anopheles* species, including *An. annularis, An. vagus, Anopheles* *nigerrimus, An. tessellatus,* and *An. barbirostris,* were also prevalent. The MHD of *An. culicifacies* in human dwellings ranged from 0 to 69, whereas in cattle sheds, it ranged from 1 to 54. For *An. fluviatilis,* the MHD ranged from 0 to 1 in human dwellings and 0 to 2 in cattle sheds. The density of *An. culicifacies* was highest between June and September, whereas the density of *An. fluviatilis* peaked during November-December.

#### Maharashtra (Gadchiroli District)

*An. culicifacies* was the predominant species, with *An. fluviatilis* and *An. stephensi* collected in negligible numbers. Other collected *Anopheles* species were *An. subpictus, An. annularis, An. vagus, An. nigerrimus, An. pallidus, An. splendidus, and An. barbirostris*. The MHD of An. culicifacies was 12.1 (range 11.5-12.7), with a significantly higher percentage found in cattle sheds than in human dwellings. *An. subpictus* and *An. culicifacies* accounted for over 95% of all anophelines, and both were perennial species, with the highest density observed in August.

#### Haryana (Nuh and Palwal Districts)

*An. culicifacies* and *An. stephensi* were primarily collected from cattle sheds. The density of these vector species was generally low but increased considerably during the postmonsoon period and the onset of winter, with an MHD ranging between 8 and 21. Among other anophelines, *An. subpictus* was found in high densities, followed by *An. annularis* and *Anopheles pulcherrimus.* The monthly MHDs of *An. culicifacies* and *An. stephensi* exhibited high variations across different seasons. Two peaks of densities of *An. culicifacies* were observed in March-April and September-October. *An. stephensi* showed the first peak from January to April and a more prominent peak from September to December. The peak densities of *An. culicifacies* were in March (48.5) and October (29.5).

#### Tripura (Dhalai and South Tripura Districts) and Meghalaya (West Garo Hills and South Garo Hills Districts)

*>An. minimus* and *An. baimaii*, the primary vectors in the states, were collected in the study districts of Tripura and Meghalaya. An. minimus was found in 9 out of the 16 selected villages, but their densities were low, especially in the state of Meghalaya. In addition to the primary vectors, *An. annularis, An. vagus, An. varuna*, and *Anopheles jeyporiensis* were among the other *Anopheles* species collected. In Tripura, the density of *Anopheles* species was higher in August, whereas in Meghalaya, it peaked in September.

#### Odisha (Angul and Kalahandi Districts)

The proportion of *An. culicifacies* and *An. fluviatilis* was higher in Kalahandi than in Angul, with an increased density of *An. culicifacies* observed in both districts. Throughout the study period, 15 and 18 different *Anopheles* species were collected in Kalahandi and Angul, respectively. The density of *An. fluviatilis* was significantly higher during winter than during the rainy and summer seasons in both districts. By contrast, the densities of *An. culicifacies* were considerably higher during the rainy season than during the postmonsoon and winter seasons.

### Anopheline Density From Spray Sheet Collections

The districts of Palwal and Nuh in Haryana exhibited the highest density of *An. culicifacies,* with 36.0 and 10.9 mosquitoes captured per room, respectively. In the remaining districts (Sidhi, Kheda, Panchmahal, Gadchiroli, and Kalaburagi), the per-room density of *An. culicifacies* ranged from 1.9 to 3.9. Similarly, collections from Palwal (45.0) and Nuh (20.4) districts showed high densities of *An. stephensi,* while in Kheda (0.02) and Panchmahal (0.06) districts in Gujarat, its density was low. *An. fluviatilis* was collected from Sidhi (Madhya Pradesh) and Kalaburagi (Karnataka) districts with a per-room density of 0.33 and 0.06, respectively.

### Anopheline Density From Light Trap Catches

The density of vectors recorded from light trap collections, that is, the number per trap night, both indoors and outdoors, is provided in Table 1. The density of *An. culicifacies* indoors and outdoors ranged from 0.4 to 3.9 mosquitoes per trap night, except in Gadchiroli, where the density was negligible. *An. stephensi* was only collected from light traps set outdoors in Palwal (8.5 mosquitoes per trap night) and Nuh (2.9 mosquitoes per trap night) districts. *An. fluviatilis* was collected from both indoor and outdoor traps in Kalaburagi, Kalahandi, and Angul, and only from indoor traps in Sidhi. In all these districts, the density of *An. fluviatilis* was below 3.8, with the exception of Kalaburagi, where it was 18.6 from indoor trap collections.

### Abdominal Condition of Vector Species in Different Collections

#### Madhya Pradesh (Sidhi District)

In Kusumu CHC, the proportion of semigravid plus gravid *An. culicifacies* in indoor human dwelling collections (hand catch and pyrethrum spray collections) was 32.8%, while in Sumaria CHC, it was 31.16%. In both CHCs, the proportion of unfed plus fully fed mosquitoes was 65.75% and 69.07%, respectively.

#### Gujarat (Kheda and Panchmahal Districts)

In Panchmahal, the proportion of unfed plus fully fed mosquitoes was higher in March, May, and June, whereas in all the remaining months, the proportion of semigravid plus gravid mosquitoes was higher, ranging from 51% to 100%. Similarly, in Kheda district, the proportion of the semigravid plus gravid category was similar to Panchmahal, except in January, October, and December. With a few monthly variations, the data suggest a predominantly endophilic behavior of *An. culicifacies* in the 2 districts.

#### Karnataka (Kalaburagi District)

In Kalaburagi district in Karnataka, the proportion of semigravid plus gravid mosquitoes in indoor human dwelling collections in all 3 villages, Laxmipurwadi (60.4%), Shankerpurwadi (81.9%), and Muglegaon (65%), was higher than unfed plus fully fed mosquitoes, suggesting that *An. culicifacies* continues to exhibit endophilic behavior. Similarly, the proportion of semigravid plus gravid *An. fluviatilis* was significantly higher than unfed plus fully fed mosquitoes in Lashmipuriwadi village, also indicating its endophilic behavior. From the collection of other villages, no conclusion can be drawn as only a few specimens of *An. fluviatilis* were collected from Muglegaon village, and in Shankerpurwadi, the collection was nil.

#### Maharashtra (Gadchiroli District)

In Gadchiroli, the proportion of semigravid plus gravid *An. culicifacies* in human dwellings (from resting and pyrethrum spray collections) was 57.6% in Ahiri CHC and 46.1% in Dhanora CHC, indicating its endophilic behavior in the district with little difference between the 2 CHCs.

### Blood Meal Preferences

**Figure 4:**
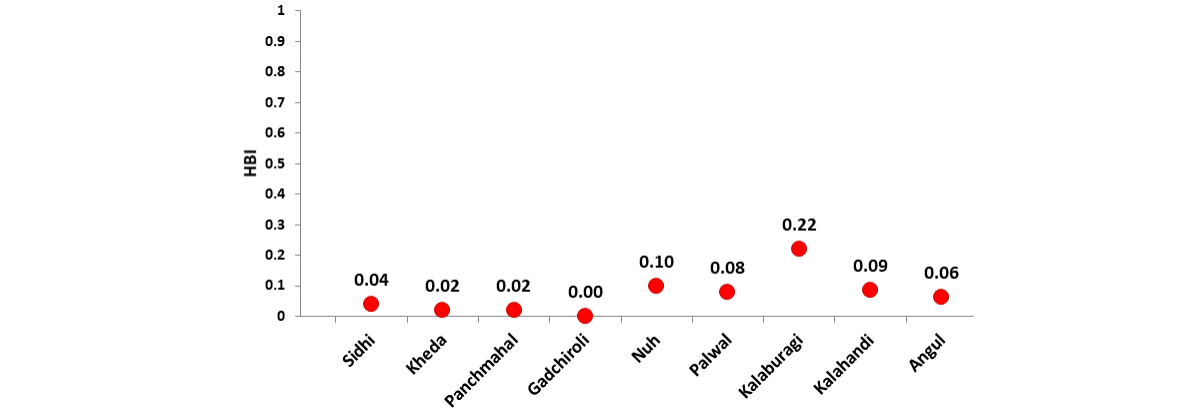
HBI of Anopheles culicifacies. HBI: Human blood index.

The HBI of *An. culicifacies* recorded in different study sites is shown in Figure 4. In Madhya Pradesh (Sidhi district), the HBI of *An. culicifacies* was 0.03 and of *An. fluviatilis* was 0.09. In Gujarat (Kheda and Panchmahal districts), the HBI of *An. culicifacies* in the canal-irrigated area of Kheda district was 0.02, whereas in the riverine area, none contained human blood. In Panchmahal district, the HBI was 0.016 in the canal-irrigated area and 0.01 in the riverine area. In Karnataka (Kalaburagi district), the HBI of *An. culicifacies* and *An. fluviatilis* was 0.22 and 0.06, respectively. In Maharashtra (Gadchiroli district), the HBI of *An. culicifacies* was 0.10 in the Gadchiroli district of Maharashtra. In Haryana (Nuh and Palwal districts), the HBI of *An. culicifacies* was low, 0.09 in Nuh and 0.07 in Palwal. Similarly, the HBI of *An. stephensi* was also low in the 2 districts, 0.01 and 0.004, respectively. In Tripura (Dhalai and South Tripura districts) and Meghalaya (West Garo Hills and South Garo Hills districts), the blood meal analysis revealed a higher HBI of 2 vector species: *An. minimus* (0.76-0.78) and *An. baimaii* (0.85), indicating their higher anthropophagic behavior in northeast India. The HBI of *An. jeyporiensis* was also found to be higher (0.66-0.75) in the same region. In Odisha (Angul and Kalahandi districts), the HBI of *An. fluviatilis* was higher in both districts, indicating that the species was primarily anthropophagic. The HBI observed in Kalahandi was 0.52, and in Angul, it was 0.32. By contrast, *An. culicifacies* was found to be primarily a zoophagic mosquito, as the observed HBI was 0.086 and 0.062, respectively, in the 2 districts.

### Sibling Species Composition

**Figure 5:**
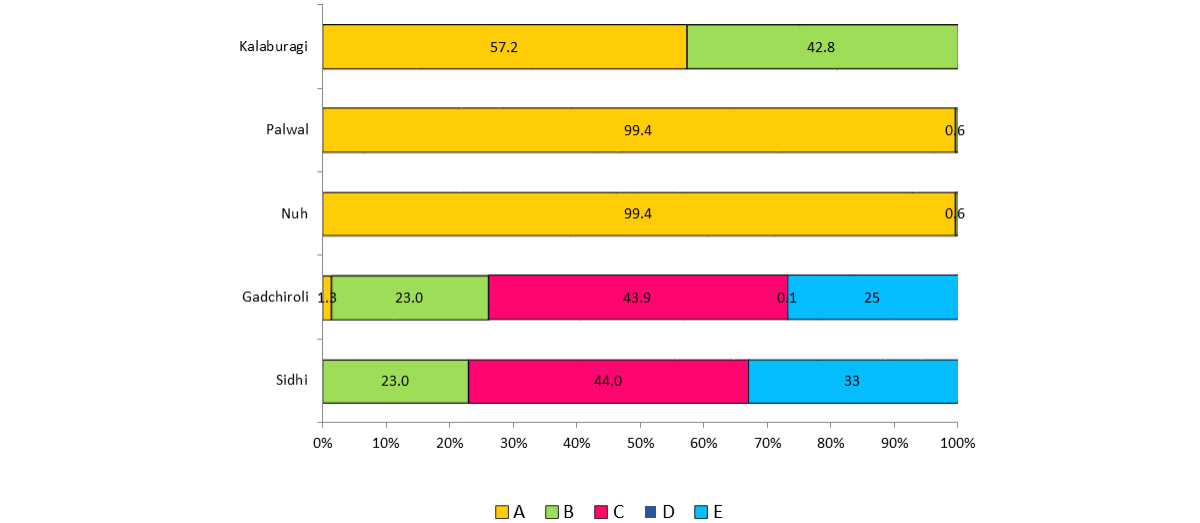
Sibling species composition of Anopheles culicifacies.

The proportion of *An. culicifacies* sibling species B, C, and E was 23%, 44.4%, and 33%, respectively, in the Sidhi district of Madhya Pradesh (Figure 5). In the Kheda district of Gujarat, sibling species A/D was 12%, and B/C/E was 82%, whereas in the Panchmahal district of the same state, the corresponding proportion was 8% and 91%, indicating the predominance of sibling species B/C/E over A/D in both districts. In the Kalaburagi district of Karnataka, *An. culicifacies* mostly comprised species A (57.2%) and species B (42.8%). The proportion of 3 sibling species S, T, and U of *An. fluviatilis* was found to be 4.6%, 94.2%, and 1.2%, respectively, in this state.

A total of 1050 specimens of *An. culicifacies* were analyzed in Maharashtra (Gadchiroli district) for the sibling species composition, where sibling species C was predominant (43.9%), followed by species E (25%). In Haryana (Nuh and Palwal districts), *An. culicifacies* species A, which is an efficient vector, predominantly constituted 99.4% in Nuh and 99.4% in Palwal.

### Vector Infection

#### Madhya Pradesh (Sidhi District)

**Figure 6:**
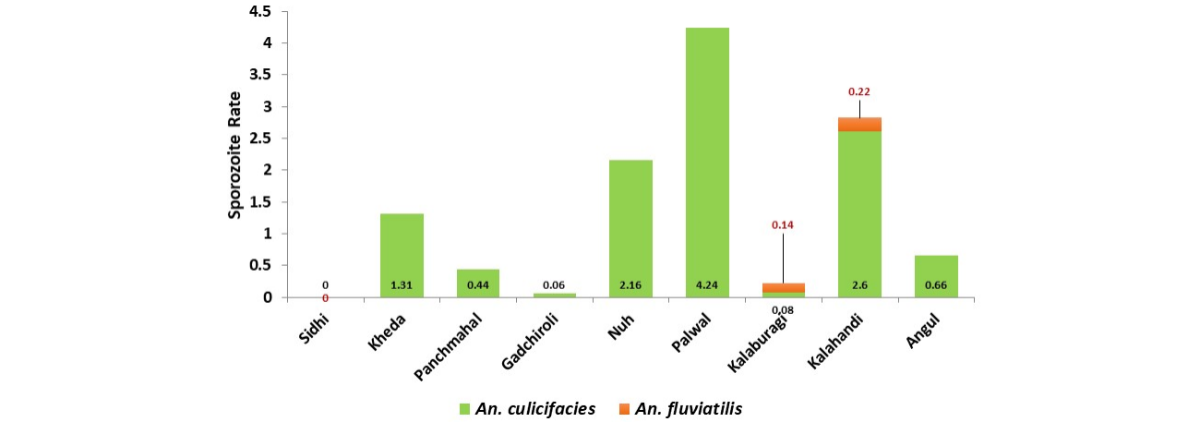
Sporozoite positivity rate (%) of Anopheles culicifacies and Anopheles fluviatilis.

A total of 1038 *An. culicifacies* and 27 *An. fluviatilis* specimens were screened for sporozoite positivity using PCR assay, and none were found positive (Figure 6).

#### Gujarat (Kheda and Panchmahal Districts)

In Kheda district, 916 *An. culicifacies* specimens were analyzed, and 12 were detected with malaria parasites (7 *P. falciparum* positive and 5. *P. vivax* positive), with a sporozoite positivity rate of 1.31%. In Panchmahal district, 4 *An. culicifacies* (3 *P. falciparum* positive and 1 *P. vivax*) specimens were found positive (n=900) with a sporozoite positivity rate of 0.44%.

#### Karnataka (Kalaburagi District)

In total, 1305 *An. culicifacies* and 691 *An. fluviatilis* specimens were screened, and the sporozoite rate was 0.08% and 0.14%, respectively.

#### Maharashtra (Gadchiroli District)

Out of 3029 *An. culicifacies* tested, only 2 were positive, showing a sporozoite positivity rate of 0.06%.

#### Haryana (Nuh and Palwal Districts)

In Nuh, 831 *An. culicifacies* specimens were analyzed for *Plasmodium* infection, and 18 were positive, with a sporozoite positivity rate of 2.16%. There was higher positivity for *P. falciparum* (n=11) than for *P. vivax* (n=7). By contrast, in Palwal district, 9 *An. culicifacies* specimens were detected positive (7 Pf positive and 2 Pv) out of 212 screened, and the sporozoite positivity rate was 4.24%.

#### Tripura (Dhalai and South Tripura Districts) and Meghalaya (West Garo Hills and South Garo Hills Districts)

None of the primary (*An. minimus* and *An. baimaii*) or secondary vectors (*An. nivipes*, *An. philippinensis,* and *An. annularis*) were found infected with malaria parasites.

#### Odisha (Angul and Kalahandi Districts)

The sporozoite rate of *An. fluviatilis* was 0.22% in Kalahandi. The sporozoite rate of *An. culicifacies* was 2.6% and 0.66% in Kalahandi and Angul districts, respectively.

### Entomological Inoculation Rate

The entomological inoculation rate of *An. culicifacies* in the Kheda and Panchmahal districts of Gujarat was 3.09 and 0.47, respectively, followed by 0.25 in the Kalaburagi district of Karnataka. The entomological inoculation rate could not be calculated for other sites.

### Vector Susceptibility to Insecticides

#### Overview

**Figure 7:**
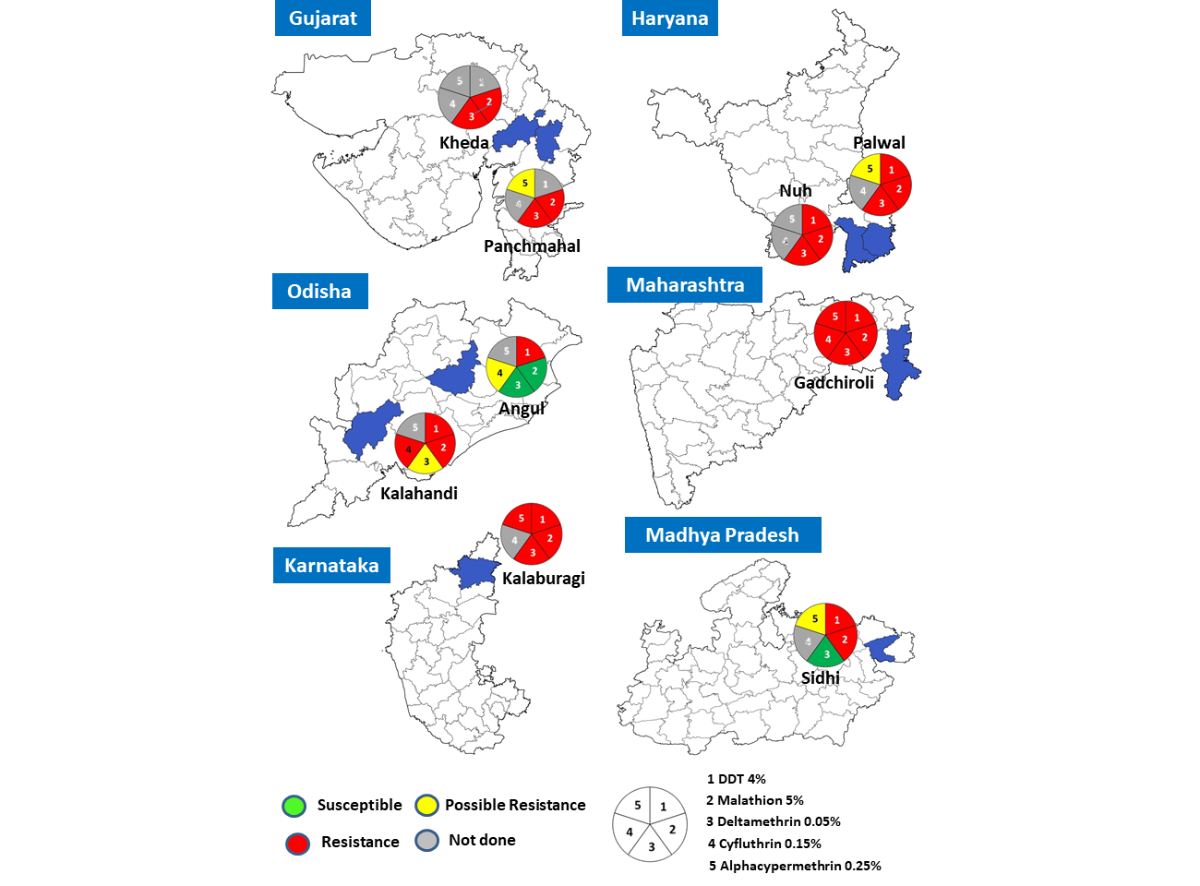
Insecticide susceptibility status of Anopheles culicifacies in India (≥98%-100% mortality: susceptible; 90-97% mortality: possible resistance; and <90% mortality: resistant). DDT: dichlorodiphenyltrichloroethane.

**Figure 8:**
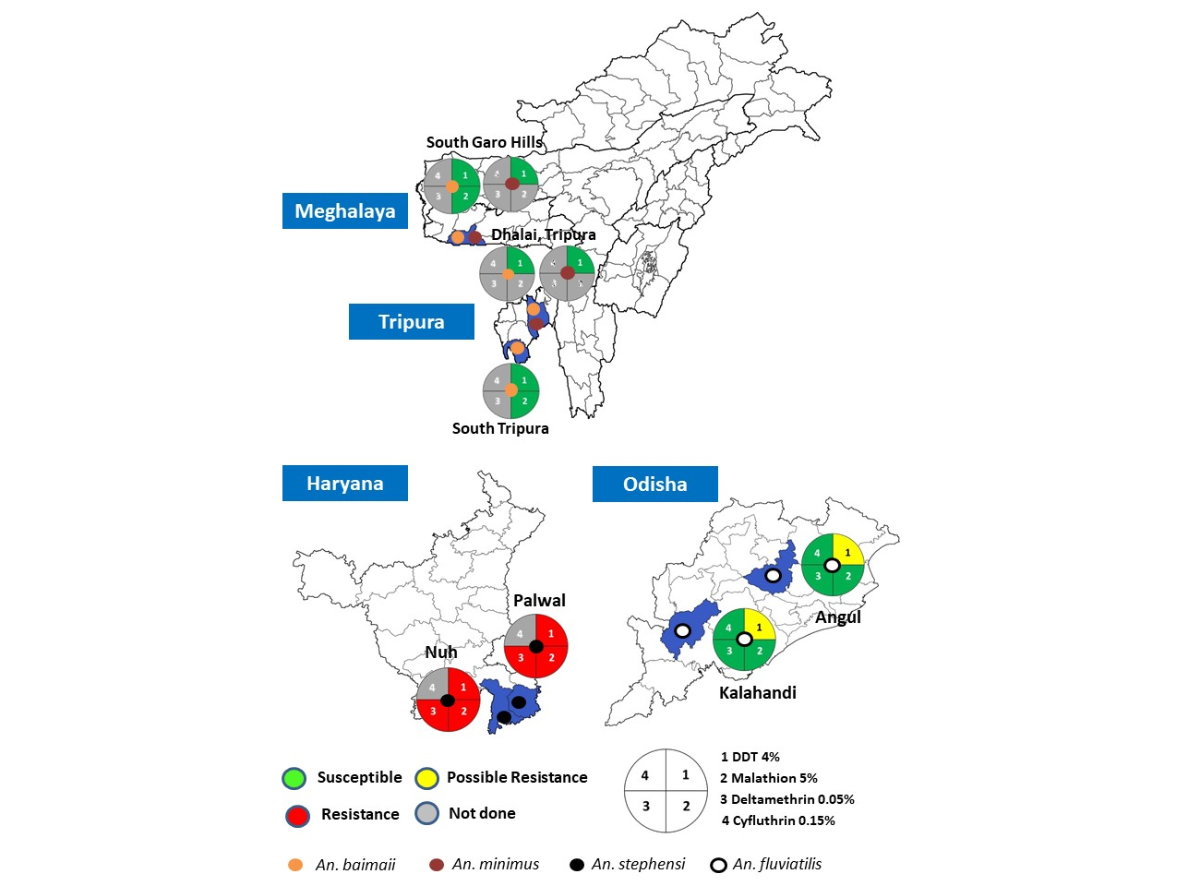
Insecticide susceptibility status of Anopheles stephensi, Anopheles fluviatilis, Anopheles minimus, and Anopheles baimaii in India (≥98-100% mortality: susceptible; 90-97% mortality: possible resistance; and <90% mortality: resistant).

Susceptibility testing was conducted on *An. culicifacies*, *An. stephensi*, *An. fluviatilis*, *An. minimus*, and *An. baimaii* collected from various study sites against DDT, malathion, and synthetic pyrethroids, including deltamethrin, cyfluthrin, and alphacypermethrin, approved by the National Malaria Control Program. The susceptibility/resistance status is visually represented in Figures 7 and 8.

#### DDT (4%)

*An. culicifacies* was tested against DDT in all districts except the Kheda and Panchmahal districts of Gujarat state, and this species exhibited resistance to DDT in all the tested districts (<39% mortality; Figure 7). *An. baimaii* and *An. minimus* were found to be susceptible to DDT in the study districts of Meghalaya and Tripura. *An. stephensi* displayed resistance to DDT in both the districts of Haryana, while *An. fluviatilis* showed possible resistance to DDT in the 2 districts of Odisha (Figure 7).

#### Malathion (5%)

*An. culicifacies* exhibited resistance to malathion (49%-78% mortality) in all districts except Angul in Odisha, where it was susceptible. *An. baimaii* was found to be susceptible to malathion in all study districts of the northeast except Dhalai, where the test could not be performed. *An. stephensi* demonstrated resistance to malathion in both districts of Haryana, whereas *An. fluviatilis* was susceptible to malathion in the 2 districts of Odisha (Figure 8).

#### Deltamethrin (0.05%)

*An. culicifacies* displayed resistance to the synthetic pyrethroid deltamethrin in Maharashtra, Gujarat, Haryana, and Karnataka study sites, but showed susceptibility in Sidhi (Madhya Pradesh) and Angul (Odisha) districts. Moreover, it exhibited possible resistance (92.5% mortality) in the Kalahandi district of Odisha. *An. stephensi* demonstrated resistance to deltamethrin in both districts of Haryana, whereas *An. fluviatilis* was found to be susceptible to this insecticide in the 2 districts of Odisha (Figure 8).

#### Cyfluthrin (0.15%)

Susceptibility tests against cyfluthrin, another synthetic pyrethroid, were conducted in Odisha and Maharashtra only. *An. culicifacies* exhibited resistance to cyfluthrin in the Gadchiroli district of Maharashtra and the Kalahandi district of Odisha, while “possible resistance” was observed in the Angul district of Odisha. *An. fluviatilis,* by contrast, was susceptible to cyfluthrin in both districts of Odisha.

#### Alpha-cypermethrin (0.25%)

Susceptibility to alpha-cypermethrin, another synthetic pyrethroid, was tested only for *An. culicifacies.* The mortality rate of *An. culicifacies* against this synthetic pyrethroid was 95%-96.7% in Panchmahal, Palwal, and Sidhi districts, indicating a possible resistance. However, in Gadchiroli (with 88.1% mortality) and Kalaburagi (with 16% mortality) districts, it exhibited resistance.

## Discussion

### Principal Findings

This study was conducted in 13 districts spanning 8 different states of India, encompassing various eco-epidemiological zones, including tribal, hilly, plain, and forested regions across the country. Notably, this is the first nationwide study covering nearly all parts of India, including the east, west, north, south, central, and northeast regions. Updated knowledge of bionomics is essential to bolster vector control activities and assess the effectiveness of such measures [22]. Therefore, the data generated in this study are crucial and highly pertinent to the country’s malaria elimination objective. Regular surveillance of malaria vectors is vital to identify any alterations in their distribution, behavior, and susceptibility to insecticides, which are essential for achieving the malaria elimination target. The findings from this study could contribute significantly to addressing these challenges.

Mosquito collections confirmed the presence of *An. culicifacies* in all study districts except those in Assam and Tripura states. The density of *An. culicifacies* obtained indoors through hand catch and pyrethrum spray sheet collection suggested that this species could be endophilic in most districts. However, the proportions of gravid and semigravid mosquitoes were lower (31%-33%) in Madhya Pradesh, indicating that *An. culicifacies* may not be entirely endophilic, and some proportions might also be resting outdoors. This could potentially indicate a shift in the resting behavior of *An. culicifacies* in Madhya Pradesh, although larger studies are necessary to confirm this possible change definitively. The alteration in resting behavior might be attributed to the use of LLINs inside houses. Similar changes in resting behavior have been reported for *An. fluviatilis* in Odisha, where it was predominantly found in mixed dwellings with a high anthropogenic nature [23]. Likewise, in Kenya and Tanzania, a shift in the resting behavior of *Anopheles gambiae s.s.* and *Anopheles funestus* was noted from endophily to exophily following the introduction of LLINs [24]. In the case of *An. stephensi,* the indoor resting density was higher than outdoors, suggesting a possible endophilic behavior as well.

*An. culicifacies,* a significant malaria vector in India, comprises a complex of 5 sibling species. It is estimated that up to about 70% of malaria transmission in India is facilitated by *An. culicifacies* [3]. Thus, the implementation of effective insecticide-based vector control interventions is crucial for malaria elimination efforts. *An. culicifacies* sibling species exhibit distinct distributions, with varying proportions of each species across different regions of the country [3], a pattern observed in this study as well. Understanding the distribution and proportions of *An. culicifacies* sibling species is crucial, as species A, C, D, and E are vectors, whereas species B is considered a poor or nonvector [25-28]. Moreover, these species vary in their susceptibility to different insecticides [28-30]. The study results indicated that species A predominated in Nuh, Palwal, and Kalaburagi districts, whereas species C was predominant in Sidhi and Gadchiroli districts. Species B was found in very low densities in Haryana, while in Gujarat and Karnataka states, its density was higher. This study offers a comprehensive overview of the contemporary distribution of sibling species in various eco-epidemiological settings across the country. Previously, the distribution pattern showed the ubiquitous presence of species B wherever *An. culicifacies* was encountered. Species A predominated in the northern part of the country, whereas species C was predominant in the western and eastern regions. In addition, species D was found in sympatric association with species A and B in the northwestern region [31].

Resistance to DDT in *An. culicifacies* was detected in all study sites, indicating widespread resistance throughout the country. Moreover, this species has developed resistance to malathion and deltamethrin in most study districts. Deltamethrin resistance in *An. culicifacies* was previously reported in the Gadchiroli district of Maharashtra [32,33], Kalahandi, and 4 other districts in Odisha [34]. Furthermore, in Nuh, Haryana, possible resistance to deltamethrin was observed [35]. A multidistrict study conducted in 2009 on the susceptibility of *An. culicifacies* to DDT, malathion, and deltamethrin in multiple districts of Madhya Pradesh reported its resistance to deltamethrin in Mandla and Dindori districts. Possible resistance was observed in Balaghat, Betul, Chhindwara, Jhabua, Sidhi, and Shadol districts, while the species remained susceptible in Guna district [36]. The national vector control program in India primarily depends on insecticide-based IRS and LLINs. Consequently, the development of resistance in malaria vectors to synthetic pyrethroids is of significant concern because of their widespread use in IRS and LLINs.

In the 1950s, DDT was used for IRS to control vectors. However, as a result of selection pressure, *An. culicifacies* was found to be resistant to this insecticide in 1959 [37]. Subsequently, malathion was introduced for vector control in 1969. However, resistance to malathion was reported in Gujarat as early as 1973 [38]. Further reports of resistance to malathion emerged from other states such as Andhra Pradesh, Odisha, Madhya Pradesh, Chhattisgarh, and Jharkhand [39]. In 1996, synthetic pyrethroids were introduced for IRS in India owing to their safety, excito-repellent, and knockdown properties. Just 6 years after their initial use, the first report of deltamethrin resistance in *An. culicifacies* emerged in Gujarat in 2002 [40]. Subsequently, triple resistance (to DDT, malathion, and deltamethrin) in *An. culicifacies* was reported from 31 districts in India [41]. The findings of this study align with these earlier reports.

*An. stephensi,* another malaria vector, was found to be resistant to deltamethrin, DDT, and malathion in the Nuh and Palwal districts of Haryana. Currently, IRS is not used as a vector control strategy for *An. stephensi* in India, except in Rajasthan, where it has been identified as the primary vector of malaria [41]. However, this species has been reported to be double resistant, specifically to DDT and malathion in 7 districts, and to malathion and deltamethrin in 1 district [41]. In our study, possibly for the first time in the country, triple resistance (to DDT, malathion, and deltamethrin) in *An. stephensi* was reported.

*An. fluviatilis* demonstrated susceptibility to malathion and synthetic pyrethroids, but exhibited possible resistance to DDT in the Kalahandi and Angul districts of Odisha. This vector species remained susceptible to deltamethrin and malathion in most parts of the country, except in Gadchiroli (Maharashtra), where possible resistance was reported [33]. In the Mayurbhanj district of Odisha, possible resistance to malathion was reported in *An. fluviatilis.* It was found to be susceptible to DDT in Odisha but resistant in Jharkhand and Chhattisgarh [41,42]. In this study, *An. minimus* and *An. baimaii,* the 2 primary malaria vectors in the northeast, were found to be susceptible to DDT and malathion. Another study by Raghavendra et al [41] reported that these 2 vector species were susceptible to DDT, malathion, and deltamethrin. However, in the eastern state of Odisha, there was only 1 report of resistance in *An. minimus* to DDT.

The WHO has proposed 3 main strategies for insecticide resistance management in malaria vectors: rotation, mosaics, and mixtures [43]. The primary objective of insecticide resistance management is to prevent the emergence of resistance in susceptible populations, delay the evolution of insecticide resistance, or reverse it to a level that allows for the effective use of insecticides for vector control [44]. Information regarding insecticide resistance among malaria vectors and the underlying mechanisms of resistance in various ecological settings is essential for formulating rational strategies for insecticide use and resistance management [45]. Therefore, further research studies on the frequency and intensity of insecticide resistance, as well as its underlying mechanisms among malaria vectors, are crucial to suggest an appropriate resistance management strategy. Using synergist piperonyl butoxide–based LLINs effectively reduced populations of insecticide-resistant (synthetic pyrethroid) vectors [46]. The ICMR-Vector Control Research Center assessed the efficacy of a piperonyl butoxide-alphacypermethrin–incorporated LLIN in experimental huts in Odisha and found it to be superior to the reference net in terms of mosquito mortality in the huts and cone bioassays [47]. Another potential method for managing insecticide-resistant populations could involve the use of attractive toxic sugar baits in combination with LLINs, although this approach is still under development and evaluation [48-50]. These proposed measures hold significant epidemiological importance for India.

We screened over 8000 anopheline mosquitoes for *Plasmodium* parasite infection, but only 2 species, *An. culicifacies* and *An. fluviatilis*, tested positive. An earlier study conducted in Nuh in 2015-2016 reported a sporozoite positivity rate of 0.26% [35], which is lower than the rate reported in this study (2.1%). The observed variability in the sporozoite rate could be attributed to differences in sample size, usage of LLINs, and malaria endemicity of the study sites. Moreover, the high positivity rate of *An. culicifacies* in Nuh and Palwal districts might be due to the predominant proportion of sibling species A (>99%) in the area. This sibling species is recognized as an established malaria vector in the malaria-endemic regions of India [35]. In a previous study conducted in 8 southern districts of Odisha, sporozoite-positive *An. culicifacies* was reported only from Kandhamal district (with a 1.5% infectivity rate), and none of the mosquito pools tested positive in the rest of the districts, including Kalahandi [51]. Regarding *An. stephensi,* none were found positive for *Plasmodium* infection in Nuh and Palwal districts. This observation might be due to the abundance of *An. stephensi* *mysorensis* form, which, being primarily zoophagic in nature, is not considered a competent vector [3]. An observed HBI of 0-0.01 in this study confirms the zoophilic behavior of *An. stephensi mysorensis* in the area.

### Limitations

This study conducted nationwide surveillance of malaria vectors, successfully collecting all primary vectors of malaria in India except *An. sundaicus,* which is the only vector in Andaman and Nicobar Islands, India. This represents a major limitation of our study. Another limitation is the unavailability of data on intensity bioassays for determining the level of insecticide resistance among the main malaria vectors. The results of intensity assays could inform decisions on whether to continue existing insecticide vector control measures or to change insecticides. The third limitation of the study was the lack of information on mosquito larval breeding habitats. This information could provide a more comprehensive understanding of the anopheline breeding habitats present in the selected study sites. The final limitation of the study was the lack of data on the parity status of vector mosquitoes, which is a robust indicator of mosquito age.

### Conclusions

This study provides a comprehensive overview of essential aspects of vector bionomics, including seasonal prevalence, resting and biting behavior, insecticide resistance status, composition of sibling species, and malaria transmission potential. It clearly indicates a growing development of resistance in *An. culicifacies* against synthetic pyrethroids in the country. These findings underscore the importance of continuous monitoring of insecticide resistance for effective planning, implementation, and evaluation of malaria vector control strategies. Furthermore, because sibling species can vary in the rate of development of insecticide resistance, monitoring should be conducted at the sibling species level rather than at the sensu lato level. The observed shift from endophilic to exophilic behavior in parts of India among *An. culicifacies* highlights the necessity for continuous monitoring of such behavioral changes in vector species, especially in light of the extensive use of LLINs in the country. The localized and focal nature of malaria transmission is influenced by variations in the biological characteristics of vector species, cultural aspects of human populations, and environmental factors within a region. Therefore, further research on vector behavior is crucial to corroborate specific findings that have implications for malaria transmission and to strengthen control measures.

## Abbreviations

DDT: dichlorodiphenyltrichloroethane
HBI: human blood index
ICMR: Indian Council of Medical Research
IRS: indoor residual spraying
LLIN: long-lasting insecticidal nets
MHD: man-hour density
PCR: polymerase chain reaction
WHO: World Health Organization

## Data Availability

The data sets generated during or analyzed in this study may be made available from the corresponding author on reasonable request.

## References

[1] World Health Organization (WHO) (2021). World Malaria Report.

[2] Malaria Situation in India. National Center for Vector Borne Diseases Control.

[3] Subbarao SK, Nanda N, Rahi M, Raghavendra K (2019). Biology and bionomics of malaria vectors in India: existing information and what more needs to be known for strategizing elimination of malaria. Malar J.

[4] National Center for Vector Borne Diseases Control (NCVBDC) (2017). National strategic plan for malaria elimination in India 2017-2022. NCVBDC.

[5] World Health Organization (WHO) (2017). Global Vector Control Response.

[6] Sharma S, Verma R, Yadav B, Kumar A, Rahi M, Sharma A (2022). What India can learn from globally successful malaria elimination programmes. BMJ Glob Health.

[7] Saxena R, Nagpal BN, Singh VP, Srivastava A, Dev V, Sharma MC, Gupta HP, Tomar AS, Sharma S, Gupta SK (2014). Impact of deforestation on known malaria vectors in Sonitpur district of Assam, India. J Vector Borne Dis.

[8] Sinka ME, Pironon S, Massey NC, Longbottom J, Hemingway J, Moyes CL, Willis KJ (2020). A new malaria vector in Africa: predicting the expansion range of and identifying the urban populations at risk. Proc Natl Acad Sci U S A.

[9] Khan N, Awasthi G, Das A (2023). How can the complex epidemiology of malaria in India impact its elimination?. Trends Parasitol.

[10] Nikookar SH, Maleki A, Fazeli-Dinan M, Shabani Kordshouli R, Enayati A (2022). Entomological surveillance of the invasive Aedes species at higher-priority entry points in northern Iran: exploratory report on a field study. JMIR Public Health Surveill.

[11] Kumar G, Gupta S, Rahi M, Sharma A (2022). Challenges in understanding the bionomics of Indian malaria vectors. Am J Trop Med Hyg.

[12] Rahi M, Anvikar A, Singh O, Jambulingam P, Vijayachari P, Das A, Pati S, Narain K, Gangakhedkar R, Dhingra N, Bhargava B (2019). MERA India: Malaria Elimination Research Alliance India. J Vector Borne Dis.

[13] World Health Organization (WHO) (1975). Manual on Practical Entomology in Malaria Part II. Methods and Techniques.

[14] Nagpal BN, Sharma VP (1995). Indian Anophelines.

[15] Goswami G, Singh O, Nanda N, Raghavendra K, Gakhar S, Subbarao S (2006). Identification of all members of the Anopheles culicifacies complex using allele-specific polymerase chain reaction assays. Am J Trop Med Hyg.

[16] Singh O, Chandra D, Nanda N, Raghavendra K, Sunil S, Sharma S, Dua V, Subbarao S (2004). Differentiation of members of the Anopheles fluviatilis species complex by an allele-specific polymerase chain reaction based on 28S ribosomal DNA sequences. Am J Trop Med Hyg.

[17] Collins R, Dash B, Agarwala R S, Dhal K B (1986). An adaptation of the gel diffusion technique for identifying the source of mosquito blood meals. Indian J Malariol.

[18] Wirtz R, Burkot T, Andre R, Rosenberg R, Collins W, Roberts D R (1985). Identification of Plasmodium vivax sporozoites in mosquitoes using an enzyme-linked immunosorbent assay. Am J Trop Med Hyg.

[19] Wirtz RA, Duncan JF, Njelesani EK, Schneider I, Brown AE, Oster CN, Were JB, Webster HK (1989). ELISA method for detecting Plasmodium falciparum circumsporozoite antibody. Bull World Health Organ.

[20] Snounou G, Pinheiro L, Gonçalves A, Fonseca L, Dias F, Brown K, do Rosario VE (1993). The importance of sensitive detection of malaria parasites in the human and insect hosts in epidemiological studies, as shown by the analysis of field samples from Guinea Bissau. Trans R Soc Trop Med Hyg.

[21] World Health Organization (WHO) (2016). Test Procedures for Insecticide Resistance Monitoring in Malaria Vector Mosquitoes, 2nd ed.

[22] World Health Organization (WHO) (1975). Manual on Practical Entomology in Malaria. Part-I: Vector Bionomics and Organisation of Antimalaria Activities.

[23] Rath A, Prusty MR, Das M, Mahapatra N, Tripathy H, Hazra RK (2015). A shift in resting habitat and feeding behavior of Anopheles fluviatilis sibling species in the Keonjhar district of Odisha, India. Trans R Soc Trop Med Hyg.

[24] Corbel V, Akogbeto M, Damien GB, Djenontin A, Chandre F, Rogier C, Moiroux N, Chabi J, Banganna B, Padonou GG, Henry M (2012). Combination of malaria vector control interventions in pyrethroid resistance area in Benin: a cluster randomised controlled trial. The Lancet Infectious Diseases.

[25] Subbarao S, Vasantha K, Raghavendra K, Sharma VG, Sharma G K (1988). Anopheles culicifacies: siblings species composition and its relationship to malaria incidence. J Am Mosq Control Assoc.

[26] Subbarao S, Adak T, Vasantha K, Joshi H, Raghvendra K, Cochrane A, Nussenzweig R, Sharma V (1988). Susceptibility of Anopheles culicifacies species A and B to Plasmodium vivax and Plasmodium falciparum as determined by immunoradiometric assay. Trans R Soc Trop Med Hyg.

[27] Kar I, Subbarao SK, Eapen A, Ravindran J, Satyanarayana TS, Raghavendra K, Nanda N, Sharma VP (1999). Evidence for a new malaria vector species, species E, within the Anopheles culicifacies complex (Diptera: Culicidae). J Med Entomol.

[28] Subbarao S K, Vasantha K, Sharma V P (1988). Responses of Anopheles culicifacies sibling species A and B to DDT and HCH in India: implications in malaria control. Med Vet Entomol.

[29] Raghavendra K, Vasantha K, Subbarao SK, Pillai MV, Sharma V P (1991). Resistance in Anopheles culicifacies sibling species B and C to malathion in Andhra Pradesh and Gujarat States, India. J Am Mosq Control Assoc.

[30] Raghavendra K, Subbarao S, Vasantha K, Pillai MV, Sharma V P (1992). Differential selection of malathion resistance in Anopheles culicifacies A and B (Diptera: Culicidae) in Haryana State, India. J Med Entomol.

[31] World Health Organization (WHO) (2007). Anopheline Species Complexes in South and South-East Asia.

[32] Chand G, Behera P, Bang A, Singh N (2017). Status of insecticide resistance in An. culicifacies in Gadchiroli (Maharashtra) India. Pathog Glob Health.

[33] Singh RK, Kumar G, Modak KS, Karlekar RR, Dhiman RC (2021). Insecticides susceptibility status of malaria vectors in a high malaria endemic tribal district Gadchiroli (Maharashtra) of India. J Commun Dis.

[34] Sahu S, Dash S, Sonia T, Gunasekaran K (2019). Synergist piperonyl butoxide enhances the efficacy of deltamethrin in deltamethrin-resistant Anopheles culicifacies sensu lato in malaria endemic districts of Odisha State, India. Indian J Med Res.

[35] Nanda N, Singh S, Prajapati B, Ranjan K, Kar N, Sharma S, Valecha N (2017). Entomological determinants of malaria transmission in an epidemic prone area of District Nuh (Haryana state), India. J Vector Borne Dis.

[36] Mishra A, Chand S, Barik T, Dua V K, Raghavendra K (2012). Insecticide resistance status in Anopheles culicifacies in Madhya Pradesh, central India. J Vector Borne Dis.

[37] Rahman J, Roy ML, Singh K (1959). Development of increased tolerance to DDT in Anopheles culicifacies Giles in the Panch Mahal district of Bombay state, India. Indian J Malariol.

[38] Rajagopal R (1977). Malathion resistance in Anopheles culicifacies in Gujarat. Indian J Med Res.

[39] Singh R, Kumar G, Mittal P (2014). Insecticide susceptibility status of malaria vectors in India: a review. Int J Mosq Res.

[40] Singh OP, Raghavendra K, Nanda N, Mittal PK, Subbarao SK (2002). Pyrethroid resistance in Anopheles culicifacies in Surat district, Gujarat, west India. Curr Sci.

[41] Raghavendra K, Velamuri PS, Verma V, Elamathi N, Barik TK, Bhatt RM, Dash AP (2017). Temporo-spatial distribution of insecticide-resistance in Indian malaria vectors in the last quarter-century: need for regular resistance monitoring and management. J Vector Borne Dis.

[42] Singh R, Dhiman R, Kumar G, Sinha AV, Dua V K (2011). Susceptibility status of malaria vectors to insecticides in Koderma, Jharkhand. J Commun Dis.

[43] World Health Organization (WHO) (2012). Global plan for insecticide resistance management in malaria vectors. WHO.

[44] Dusfour I, Vontas J, David J, Weetman D, Fonseca DM, Corbel V, Raghavendra K, Coulibaly MB, Martins AJ, Kasai S, Chandre F (2019). Management of insecticide resistance in the major Aedes vectors of arboviruses: advances and challenges. PLoS Negl Trop Dis.

[45] Munywoki DN, Kokwaro ED, Mwangangi JM, Muturi EJ, Mbogo CM (2021). Insecticide resistance status in Anopheles gambiae (s.l.) in coastal Kenya. Parasit Vectors.

[46] Killeen G (2014). Characterizing, controlling and eliminating residual malaria transmission. Malar J.

[47] Kasinathan G, Sahu SS, Tharmalingam V, Swaminathan S, Rahi M, Purushothaman J (2019). Evaluation of MAGNet, a long-lasting insecticidal mosquito net against Anopheles fluviatilis in experimental huts in India. Malar J.

[48] Stewart ZP, Oxborough RM, Tungu PK, Kirby MJ, Rowland MW, Irish SR (2013). Indoor application of attractive toxic sugar bait (ATSB) in combination with mosquito nets for control of pyrethroid-resistant mosquitoes. PLoS One.

[49] Kumar G, Ojha VP, Pasi S (2021). Applicability of attractive toxic sugar baits as a mosquito vector control tool in the context of India: a review. Pest Manag Sci.

[50] Kumar G, Sharma A, Dhiman R (2022). Laboratory evaluation of the efficacy of boric acid containing toxic sugar baits against and mosquitoes. J Vector Borne Dis.

[51] Sahu S, Gunasekaran K, Krishnamoorthy N, Vanamail P, Mathivanan A, Manonmani A, Jambulingam P (2017). Bionomics of Anopheles fluviatilis and Anopheles culicifacies (Diptera: Culicidae) in relation to malaria transmission in East-Central India. J Med Entomol.

